# Course of Study for Trained Hospital Nurses

**Published:** 1922-09

**Authors:** 


### COURSE OF STUDY FOR TRAINED HOSPITAL NURSES.

### To qualify for positions under Local Health Authorities (approved by the Scottish Board of Health).

In order to meet the requirements laid down in the regulations issued by the Scottish Board of Health for the training of health visitors, a special course of study has been arranged by the Edinburgh School of Social Study and Training, under the auspices of the University. Students who successfully complete this course and gain the certificate of the Central Midwives Board will be awarded the Health Visitor's Probation Certificate. The course is arranged to cover a period of seven months, and students may commence either in Oct., 1922, or in Jan., 1923.

PROBATION NURSES.

The course detailed should provide considerable help to those who, in the future, intend to take up nursing as a profession. The regulations of the Scottish Board of Health make it possible for the future health visitor to enter for the course qualifying for the Health Visitor's Probation Certificate either before or after hospital training. Lectures marked* cover the syllabus in the subjects laid down by the General Nursing Council for Scotland.

SANITARY SCIENCE.

A course of 50 lectures by Mr. Allan Ritchie, M.R.S.I., is also arranged, covering the syllabus of the Sanitary Association of Scotland. This class meets on Tuesday and Thursday, at 6 p.m., in the autumn term, and on Tuesday, Thursday, and Friday, at 6 p.m., in the spring term. Fee, 30s. each term.

CURRICULUM.

Elementary Anatomy and Physiology.*—Mr. F. E. JARDINE. 30 lectures, Tuesday and Thursdays, at 5 p.m., in autumn and first half of spring term.

General Hygiene.*—Dr. W. ROBERTSON. 20 lectures, Mondays and Wednesdays, at 4 p.m., in the autumn term.

Hygiene of Childhood.—Dr. T. Y. FINLAY. 10 lectures, Mondays, at 5 p.m., in the spring and summer terms.

School Hygiene.—Dr. HALLY MEIKLE. 10 lectures, Tesdays, at 4 p.m., in the summer term.

Local Government.—Lady LESLIE MACKENZIE. Mondays and Wednesdays, at 4.30 p.m. Spring term.

Social Economics.—Miss NORA MILNES, B.Sc. 30 lectures, Wednesdays, at 6 p.m., and Fridays, at 5 p.m., in the autumn and spring terms.

Social Psychology.—Dr. JAMES DREVER. 20 lectures, Fridays, at 8 p.m., in the autumn and spring terms. (Lectures arranged by the W.E.A.)

Office Work, including Indexing and Filing.—Miss E. T. M'KNIGHT, M.A. Mondays, at 4 p.m., autumn term.

Dietetics and Cooking.—10 classes. Hour to be arranged.

Each student will be required to devote half her time to practical work—*e.g.*, attendance at child welfare clinics, home visiting, visiting institutions, &c. A nurse may be excused any part of the course provided she can satisfy the authorities that she holds certificates which should justify the exemption.

FEES.—For the entire course, exclusive of the class on Dietetics and Cooking, 8 guineas. Any nurse who is exempted from part of the course will have her fees correspondingly reduced. For any individual class meeting twice a week, 30s. each term. For any individual class meeting once a week, 15s. each term.

---

The National Health Society, in compliance with the amendment to the regulations of the Board of Education on the training of health visitors, is arranging a shortened course of training for fully-qualified nurses and health visitors of three years' service under local authorities. The next course begins in September. Particulars may be obtained from the Secretary, 53 Berners Street, W.1.

---

The first prescribed examination under the Dentists' Act (1921) was held at Manchester on July 11-12 last. Of 65 candidates presenting themselves, 45 satisfied the examiners and may now be eligible for registration as dentists. Of the 15 war-service candidates, four satisfied the examiners in Part I. only, and one in Part II. of the examination.

